# *De Novo* Assembly and Characterization of *Sophora japonica* Transcriptome Using RNA-seq

**DOI:** 10.1155/2014/750961

**Published:** 2014-01-02

**Authors:** Liucun Zhu, Ying Zhang, Wenna Guo, Xin-Jian Xu, Qiang Wang

**Affiliations:** ^1^Institute of System Biology, Shanghai University, Shanghai 200444, China; ^2^Yangzhou Breeding Biological Agriculture Technology Co. Ltd., Yangzhou 225200, China; ^3^Department of Mathematics, Shanghai University, Shanghai 200444, China; ^4^State Key Laboratory of Pharmaceutical Biotechnology, School of Life Sciences, Nanjing University, Nanjing 210093, China

## Abstract

*Sophora japonica* Linn (Chinese Scholar Tree) is a shrub species belonging to the subfamily Faboideae of the pea family Fabaceae. In this study, RNA sequencing of *S. japonica* transcriptome was performed to produce large expression datasets for functional genomic analysis. Approximate 86.1 million high-quality clean reads were generated and assembled *de novo* into 143010 unique transcripts and 57614 unigenes. The average length of unigenes was 901 bps with an N50 of 545 bps. Four public databases, including the NCBI nonredundant protein (NR), Swiss-Prot, Kyoto Encyclopedia of Genes and Genomes (KEGG), and the Cluster of Orthologous Groups (COG), were used to annotate unigenes through NCBI BLAST procedure. A total of 27541 of 57614 unigenes (47.8%) were annotated for gene descriptions, conserved protein domains, or gene ontology. Moreover, an interaction network of unigenes in *S. japonica* was predicted based on known protein-protein interactions of putative orthologs of well-studied plant genomes. The transcriptome data of *S. japonica* reported here represents first genome-scale investigation of gene expressions in Faboideae plants. We expect that our study will provide a useful resource for further studies on gene expression, genomics, functional genomics, and protein-protein interaction in *S. japonica*.

## 1. Introduction

*Sophora japonica *Linn (Chinese Scholar Tree) is a shrub of the pea family Fabaceae. It grows into a lofty tree 10–20 m tall that produces a fine, dark brown timber. It is not only a kind of popular ornamental tree, but also a valuable nectar tree, offering delicious and healthy food. Moreover, dried flowers and buds of *Sophora japonica*, containing many kinds of components such as flavones, tetraglycosides, isoflavones, and isoflavone tetraglycosides [1], are used as useful herb to treat hemorrhoids and hematemesis in China, Japan, and Korea [2]. In spite of its medicinal and economic value, not much genomic or transcriptomic information is available for *S. japonica*. As of September 2013, only 74 nucleotide sequences and 35 proteins from *S. japonica* were available in GenBank. Hence, generation of genomic and transcriptome data is necessary to help further studies on *S. japonica*.

In the latest decade, the emergence of the next generation sequencing (NGS) technology offers a fast and effective way for generation of transcriptomic datasets in nonmodel species using various platforms such as Roche 454, Illumina HiSeq, and Applied Biosystems SOLiD [3–5]. Compared to the whole-genome sequencing, RNA-seq, which is considered as a cost-effective and ultra-high-throughput DNA sequencing technology, is a revolutionary advance in the functional genomic research [6]. In this approach, sequences of the expressed parts of the genome are produced [7] to identify genes [8] and explore the low abundance transcripts [9]. Due to the many advantages, RNA-seq is specifically attractive for nonmodel organisms without genomic sequences [10–13].

In this study, we used RNA-seq technology to investigate the transcriptome of S. *japonica* from three tissues. Using Illumina sequencing platform, a total of 86139654 reads of S. *japonica* transcriptome were produced. Those were assembled into 57614 unigenes and annotated for functionality. Furthermore, the protein-protein interaction network of expressed genes in S. *japonica* was constructed. This is the first S. *japonica* and *Styphnolobium* genus transcriptome data generated by RNA-seq technology. The information provides a good resource for further gene expression, genomics, and functional studies in S. *japonica*.

## 2. Method

### 2.1. RNA Preparation and Sequencing

*S. japonica *(provided by the Yangzhou eight strange Memorial) was grown in an open-air place in Jiangsu Province, Eastern China. Total RNA was extracted using TRIzol method (Invitrogen) from three different tissues: tender shoots, young leaves, and flower buds. RNA was isolated from every tissue and mixed together in equal proportion for cDNA preparation.

The poly-A mRNA was isolated from the total RNA using poly-T oligo-attached magnetic beads (Illumina). After purification, fragmentation buffer (Ambion, Austin, TX) was added to digest the mRNA to produce small fragments. These small fragments were used as templates to synthesize the first-strand cDNA with superscript II (Invitrogen) and random hexamer primers. The synthesis of the second strand was performed in a solution containing the reaction buffer, dNTP, RNaseH, and DNA polymerase I using Truseq RNA sample preparation Kit. Next, these cDNA fragments were handled with end repair using T4 DNA polymerase, Klenow DNA polymerase, and T4 polynucleotide kinase (Invitrogen). Illumina's paired-end adapters were then ligated to the two ends of cDNA fragments. The adapter sequences were as follows: read1 adapter: AGATCGGAAGAGCACACGTC and read2 adapter: AGATCGGAAGAGCGTCGTGT. The products from this ligation reaction were electrophoresed on a 2% (w/v) agarose gel (certified low range ultragrade agarose from Bio-Rad) and purified according to appropriate size of DNA fragments suitable for Illumina sequencing. Then the sequencing library was constructed according to the protocol of the Paired-End Sample Preparation kit (Illumina). Sequencing was done with an Illumina Hiseq 2000. Raw read sequences are available in the Short Read Archive database from National Center for Biotechnology Information (NCBI) with the accession number SRR964825.

### 2.2. *De Novo* Assembly

After removal of adaptor sequences along with low quality reads and reads of larger than 5% unknown sequences, the resting were assembled into unitranscripts and unigenes by Trinity [14].

We used RSEM [15] to quantify expression levels of each unique transcript (see additional file 1 in Supplementary Material available online at http://dx.doi.org/10.1155/2014/750961). Results were reported in units of TPM (transcripts per million). After counting fraction of each isoform, we used length × isoform percent as a standard to choose unigenes (see additional file 2).

### 2.3. Functional Annotation and Classification

All assembled unigenes, longer than 300 bps, were further analyzed to predict putative gene descriptions, conserved domains, gene ontology (GO) terms, and association with metabolic pathways. First of all, all the unigenes were searched in the protein databases including NCBI NR, Swiss-Prot, and clusters of orthologous groups (COG) [16] through BLASTALL procedure (ftp://ftp.ncbi.nih.gov/blast/executables/release/2.2.18/) with an *E*-value < 1.0*E* − 6. After obtaining the features of the best BLASTX hits from the alignments, putative gene names and “CDS” (coding DNA sequences) were determined. Subsequently, according to the NR annotation, we took advantage of Blast2GO [17] software to predict GO terms of molecular function, cellular component, and biological process. After obtaining GO annotation for all unigenes, GO functional classification of the unigenes performed using WEGO software [18] and exhibited the distribution of gene functions at the second level. Unigene sequences were also compared to the COG database to predict and classify possible gene functions based on orthologies. Association of unigenes with the KEGG pathways was determined using BLASTX against the Kyoto Encyclopedia of Genes and Genomes database [19]. The KEGG pathways annotation was performed in the KEGG Automatic Annotation Server (KAAS) (http:/www.genome.jp/tools/kaas/) [20].

To obtain the potential protein coding sequences from all unigenes, we first predicted all the open reading frames (ORFs). According to the BLASTP results against NR database, we chose the correct ORFs as potential protein coding sequences. And the longest ORFs from the unigenes without BLASTP results were considered as referential protein coding sequences (additional file 3).

### 2.4. Construction and Topological Analysis of Protein Interaction Network

The interaction network of unigenes in *S. japonica* was constructed in the form of nodes and edges where nodes represent genes and edges represent interactions between genes. First, we downloaded protein-protein interactions (PPI) and sequences of six species *Arabidopsis thaliana, Arabidopsis lyrata, Oryza sativa *subsp. *Japonica, Brachypodium distachyon, Populus trichocarpa*, and *Sorghum bicolor* from STRING database that is a precomputed database for the detection of protein-protein interactions [21]. Then, the protein sequences of genes from PPIs were searched against the unigenes datasets in our study to find homologies by TBLASTN (*E*-value < 1.0*E* − 6). The TBLASTN hits with identity >50% and covering query gene >80% were identified as the candidate interacting genes of the network. According to the known PPI network of the above six species, the interaction network of *S. japonica *was constructed using the homologous unigenes from the TBLASTN searches.

The topological features such as the degree distribution of nodes, degree correlation, clustering coefficient (*C*), and shortest path length (*L*) were determined for the resultant networks. To each node *i* of the network, we assigned a degree *k*_*i*_, which is the number of its neighbors. We calculated the degree distribution of the giant component (i.e., the probability *P*(*k*) that a protein has *k* edges) [22] using the equation

(1) $$\begin{array}{c} P\left(k\right)=\frac{N\left(k\right)}{N}, \end{array}$$

where *N* is the number of nodes and *N*(*k*) is the number of nodes with degree *k*.

The degree correlation, which is characterized by analyzing the average degree of nearest neighbors *k*_*nn*,*i*_ [23], is defined by

(2) $$\begin{array}{c} k_{nn,i}=\frac{1}{k_{i}}\sum_{j}a_{ij}k_{j}. \end{array}$$

The clustering coefficient (*C*) was defined as the average probability with which two neighbors of a node were also neighbors to each other. For instance, if a node *i* had *k*_*i*_ links, and among its *k*_*i*_ nearest neighbors there were *e*_*i*_ links, then the clustering coefficient of *i* [23] was calculated using the equation

(3) $$\begin{array}{c} C_{i}=\frac{2e_{i}}{k_{i}\left(k_{i}-1\right)}. \end{array}$$

The shortest path length (*L*) between two nodes was defined as the minimum number of intermediate nodes that must be traversed to go from one node to another [23]. The average shortest path length was the shortest path length averaged over all the possible pairs of nodes in the network.

## 3. Result

### 3.1. *De Novo* Sequence Assembly of *S. japonica* Transcriptome

Total RNA from three different tissues (tender shoots, young leaves, and flower buds) was extracted and blended in equal proportions for Illumina sequencing. A total of 86.1 million high-quality clean reads with total of 8700105054 nucleotides (nt) sequences were produced with an average length of 101 bps for each short read (Table 1).

**Figure 1:**
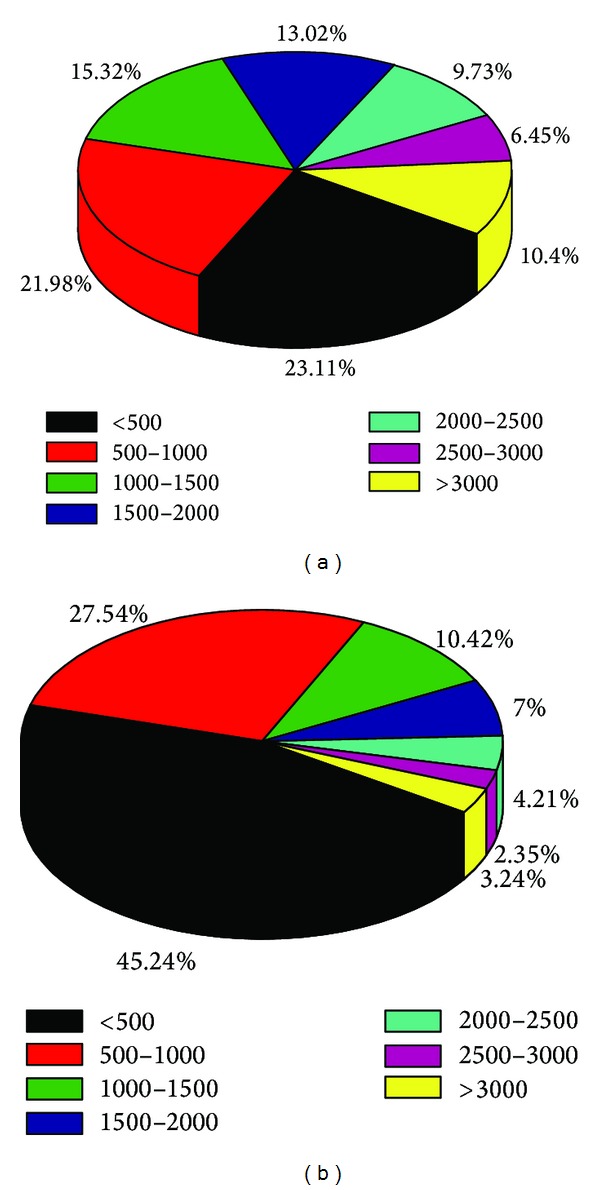
Overview of the *S. japonica* transcriptome assembly shown by pie graphs. The size distribution of the UTs (a) and unigenes (b) produced from *de novo* assembly of reads by trinity.

As a result of the absence of the genomic sequences of *S. japonica*, the transcripts were assembled *de novo* from all high-quality reads by Trinity [14]. A total of 143010 unique transcripts (UTs) were predicted from the clean sequence reads, with an average length of 1482 bps and an N50 of 1155 bps. The majority of UTs (33045) were between 100 and 500 bps, which accounted for 23.1% of total UTs shown in Figure 1(a). Then after removing redundancy, 57614 unigenes were generated with an average length of 901 bps. As shown in Figure 1(b), the length of the unigenes ranged from 300 bps to more than 3000 bps.

The quality score distribution across all bases and over all sequences was shown in additional files 4 and 5, revealing that most of the sequences have quality score larger than 30. To further evaluate the quality of the dataset, we compared the unigenes from* S. japonica* with other species using BLASTX   (additional file 6). The result showed that more than half of unigenes that are having significant BLAST hits were mapped to soybean, which was consistent with our expectation.

### 3.2. Functional Annotation and Classification of *S. japonica* Transcriptome

**Figure 2:**
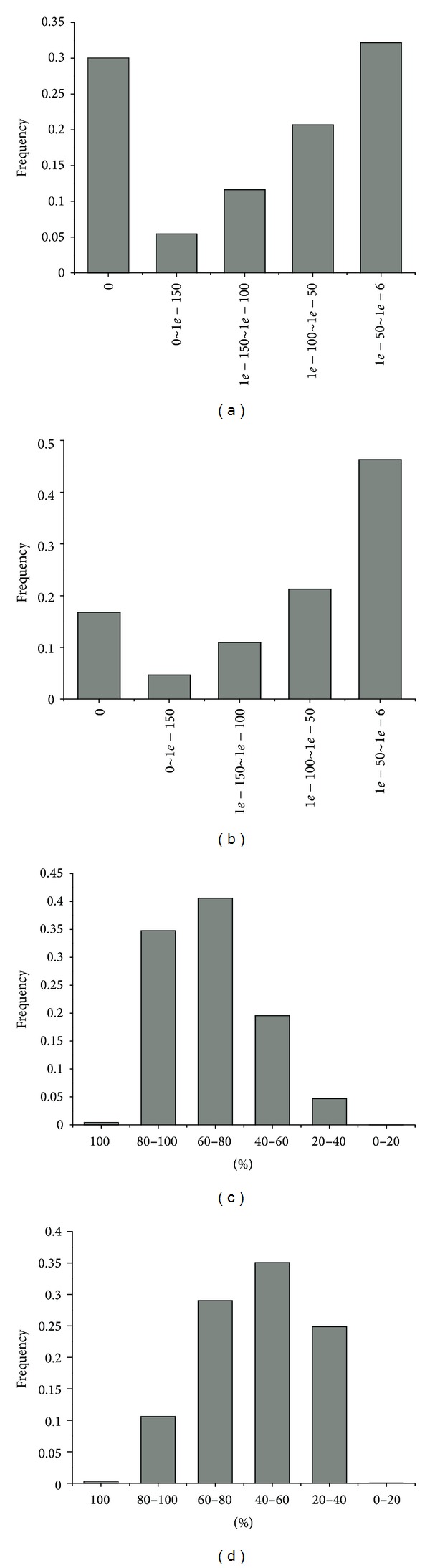
Unigene homology searches against NR and Swiss-prot databases. *E*-values proportional frequency distribution of BLAST hits against the NR database (a) and Swiss-prot database (c). Proportional frequency distribution of unigenes similarities against the NR database (b) and Swiss-Prot database (d) based on the best BLAST hits (*E*-value ≤ 1.0*E* − 5).

In order to annotate the transcriptome of *S. japonica*, a total of 57614 unigenes were first examined against the NR database in NCBI using BLASTX with an *E*-value cut-off of 1*e* ^−6^, which showed 27507 (47.7%) having significant BLAST hits (Table 2). The *E*-value distribution of significant hits revealed that 67.8% of matched sequences had strong homology (smaller than 1.0*e* − 50), while the other homologous sequences (32.2%) had *E*-values in the range of 1.0*E* − 50–1.0*E* − 6 (Figure 2(a)). The distribution of sequence similarities represented that most of the BLASTX hits (95.3%) were in the range between 40% and 100%. Only 4.7% of hits had sequence similarity values less than 40% (Figure 2(b)).

The protein coding sequences of unigenes were also compared with the protein database at Swiss-Prot by BLASTX. A total of 20463 of 57614 unigenes (35.5%) showed hits at an *E*-value threshold of ≤1.0*E* − 6 (Table 2). More than half of the matched sequences (53.7%) had strong homologies with *E*-values of ≤1.0*E* − 50, and the remaining unigenes had *E*-values between 1.0*E* − 50 and 1.0*E* − 6 (Figure 2(c)). The distribution of sequence similarities against Swiss-Prot was different than that obtained against the NR database. While 75.0% of query sequences against Swiss-Prot had similarities between 40% and 100%, only 25.0% of sequences had strong homologies with <40% identity (Figure 2(d)). Thus by combining the results of sequence similarity searches from NR and Swiss-Prot database, we identified a final set of 27541 unigenes.

### 3.3. Gene Ontology (GO) Classification

**Figure 3:**
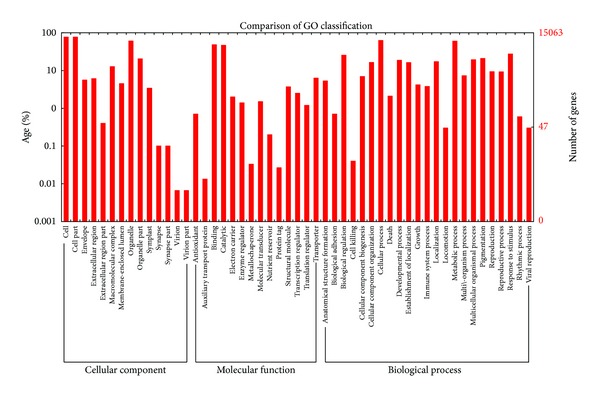
Gene ontology classification of the *S. japonica* transcriptome. Gene ontology (GO) terms associated with *S. japonica* unigenes based on significant hits against the NR database. They are summarized into three main GO categories (biological process, cellular component, and molecular function) and 51 subcategories.

GO terms were predicted for each assembled unigene to characterize functionality of gene products on the basis of their sequence similarities to known proteins in the Nr database. Of the 57614 unigenes of *S. japonica*, a total of 15063 unigenes were assigned to at least one of the three main GO categories: cellular component (11860, 20.6%), biological process (11643, 20.2%), and molecular function (11160, 19.4%). These GO terms were further subdivided into 51 sub-categories (Table 2, Figure 3, and additional file 7). Among these categories, the “cell,” “cell part,” “cellular process,” “organelle,” “metabolic process,” “catalytic activity,” and “binding” terms were found to have association with relatively more number of unigenes than other GO terms. The relative abundance of unigenes associated with cellular processes (9396) and metabolic processes (9010) in the biological processes category implied that the *S. japonica* tissues used in the study processed extensive metabolic activities.

### 3.4. COG Classification

**Figure 4:**
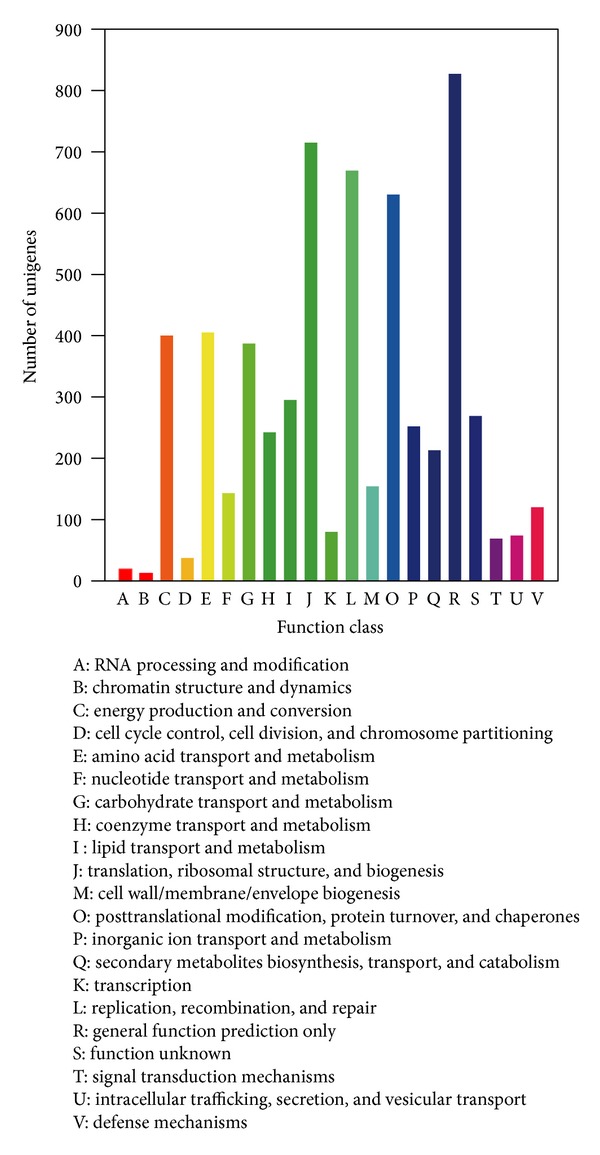
COG functional classification of the *S. japonica* transcriptome. Of 57614 unigenes in the NR database, 5863 unigenes show significant homologies to the COGs database (*E*-value ≤ 10−6) which were classified into 21 COG categories.

Cluster of Orthologous Groups (COG) database was used to classify the predicted proteins based on orthologous relationships of deduced amino sequences with 66 genomes, including bacteria, plants, and animals. Only individual proteins or groups of paralogs from at least three lineages involved in each COG were considered to be an ancient conserved domain. A total of 5863 *S. japonica* unigenes (10.2% of all unigenes) showed significant homology in the COG database. Since some of these unigenes were annotated with multiple COG functions, a total of 6012 functional annotations were predicted (*E*-value ≤1.0*E* − 6). Those were mapped to 21 COG clusters (Table 2, Figure 4, and additional file 8). The top five categories based on number of orthologies were (1) “general function prediction only” (13.8%); (2) “translation, ribosomal structure, and biogenesis” (11.9%); (3) “replication, recombination, and repair” (11.1%); (4) “posttranslational modification, protein turnover, and chaperones” (10.5%); and (5) “amino acid transport and metabolism” (6.7%). The two categories comprising “RNA processing and modification” and “chromatin structure and dynamics” consisted of 19 and 12 unigenes (0.5%), respectively, representing the two small COG classifications.

### 3.5. KEGG Pathway Mapping

To further predict the metabolic pathway in *S. japonica, *the assembled unigenes were annotated with corresponding enzyme commission (EC) numbers in the KAAS using *Arabidopsis thaliana* and *Oryza sativa* as references. A total of 2869 unigenes were mapped to 309 pathways corresponding to six KEGG modules: metabolism, genetic information processing, environmental information processing, cellular processes, and organismal systems and human diseases (additional file 9). Metabolic pathways had the largest number of unigenes (2155 members, 47.2%), followed by ribosome (158 members, 5.5%, ko03010), biosynthesis of amino acids (139 members, 4.8%, ko01230), carbon metabolism (130 members, 4.5%, ko01200), spliceosome (129 members, 4.5%, ko03040), protein processing in the endoplasmic reticulum (123 members, 4.3%, ko04141), plant hormone signal transduction (122 members, 4.3%, ko04075), purine metabolism (107 members, 3.7%, ko00230), and RNA transport (100 members, 3.5%, ko03013).

In conclusion, 27541 unigenes were annotated using NR, Swiss-Prot, COG, and KEGG databases. These unigenes had BLASTX scores with *E*-values ≤ 1.0*E* − 6. Among these, 1561 unigenes showed hits in all the four public databases (NR, Swiss-Prot, COG, and KEGG) providing the best functional annotations of those unigenes (Table 2). These annotations provide a valuable resource to investigate further processes, structures, functions, and pathways of *S. japonica* in future studies.

### 3.6. Construction and Topological Analysis of Protein-Protein Interaction Network in *S. japonica*

**Figure 5:**
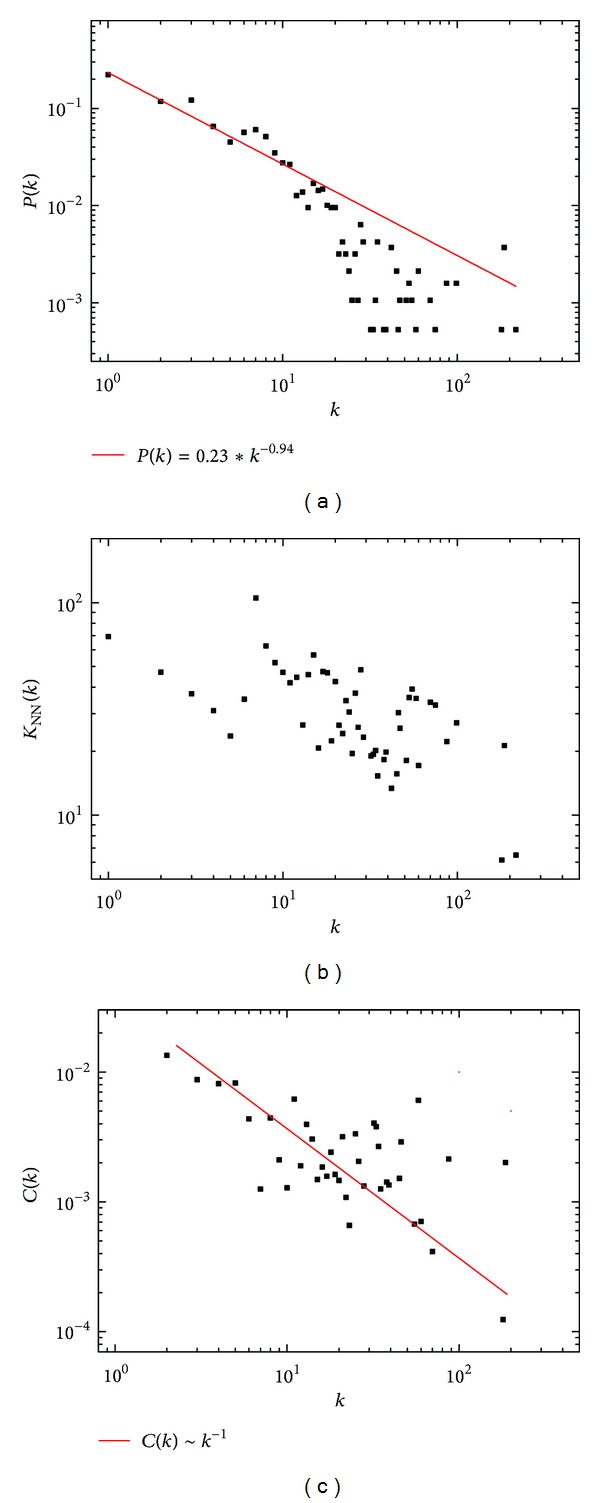
The topological analysis of the giant component of *S. japonica* protein interaction with 1887 nodes and 7634 edges. (a) Log-log plots of the node degree distribution with a power-law fit (red line). (b) Average nearest-neighbor degree *k*_*nn*_ as a function of the node degree *k*. (c) Log-log plots of the average clustering coefficient *C* as a function *k* with a guideline *C*(*k*)~*k* ^−1^ (red line).

The interaction network was constructed using the annotated unigenes of *S. japonica* in comparison with genes with at least one known protein-protein association and links of the six genomes in STRING database (additional file 10 and additional file 11). The network included one giant component and 88 small components. The giant component consisted of 1887 nodes connected via 7634 edges.  Figure 5(a) showed the degree distribution *P*(*k*) = 0.23 K^−0.94^ (the least square fit of associations) which implies the sale-free characteristics of the component. The degree-correlation of the giant component is shown in Figure 5(b). The decay behavior of *k* *nn* with *k* suggests the disassortative mixing of nodes. We plotted the clustering coefficient of a node with *k* links in Figure 5(c) yet. The guideline is *C*(*k*) ~ *k* ^−1^, the scaling law which reflects the hierarchy of the giant component. Besides, we compared the clustering coefficient *C* and the shortest path length *L* of the giant component with *Erdős-Rényi*, *Watts-Strogatz,* and *Barabási-Albert* models of the same nodes *N* and links *E*. The data in Table 3 shows high clustering coefficient and small path length. Those suggest the small-world properties of the network.

## 4. Discussion

*Sophora japonica Linn* is an economically important species for several reasons. It is commonly used to afforest cites and highways for their adaptability to ecology and environment. It also provides useful products such as honey and lumber for human use [2]. Apart from such ecological and economical values of pagoda tree, it has a unique mythological importance to Chinese people. The pagoda tree mentioned in the famous Chinese idiom story “A Fond Dream of Nanke” is believed to still present in the yard of the Yangzhou eight strange Memorial at present, in Jiangsu Province. This story tells that more than one thousand years ago, a person named Nanke drunk and rested against the pagoda tree having a dream. In his dream, he became the prime minister of the kingdom of pagoda tree. After waking up, he found that the kingdom of pagoda tree was the nest of ants under the pagoda tree. Nowadays, people believe that the tree is over 2000 years old. However, very little research has been done with this important species to understand its genome. Recently, high-throughput RNA sequencing has offered a new avenue to generate abundant sequence information from any organism [24, 25]. The data obtained from RNA-seq projects are also helpful in inferring the basic biological, molecular, and cellular processes [19, 20]. Genomes of many plant species have been studied by *de novo* transcriptome analysis, such as willow [26], *Cocos nucifera *[27], tea plant [10], and pineapple [28]. In this study, we used Illumina RNA-seq technology to sequence the *Sophora japonica *plant transcriptome and predicted a large number of expressed genes in *S. japonica*. We obtained 8.7 G bps coverage with 86.1 million high-quality clean reads. Using *de novo* software Trinity, we generated 57614 unigenes. Our results revealed that 27541 unigenes (47.8% of all assembled unigenes) were functionally annotated and involved in different biological processes.

Using the sequences of the predicted unigenes, we constructed a protein-protein interaction network to understand gene interactions in *S. japonica*. We identified a giant and 88 small components of the network. The best fit of the degree distribution of the giant component demonstrated that it was a scale-free network in which a few proteins interacted with high connectivity [29]. Like other biological networks, the giant component displays disassortative mixing that ensures connection of high-degree nodes with low-degree nodes. It is likely that the disassortativity may reduce the proportion of the important edges among hubs and increase the stability of biological networks when compared to the assortative network.

In addition, the giant component of the network also exhibited small-world properties including the high clustering coefficient as well as the smaller and shortest path length (Table 3), suggesting that the neighbors of one node have close associations among each other in the network. The smaller shortest path length is an indicative of minimal distance between a node and its target to minimize energy involved in interactions between proteins. At the same time, the scaling law of the average clustering coefficient as a function of the degree (Figure 5(c)) indicates the hierarchical structure which reflects the evolutionary patterns associated with various organizational levels of the network. The combination of many local variations, which affect the small but highly interacted nodes, slowly affects the properties of the larger but less interacted nodes [30]. Such a process during evolution ensures both stability and low energy consumption in an efficient protein-protein interaction.

## 5. Conclusion

In this study, we applied Illumina RNA sequencing and *de novo* assembly approach to study the *S. japonica* transcriptome for the first time. Totally, about 86.1 million reads assembled into 57614 unigenes were generated with an average length of 1321 bps. Among these unigenes, 27541 unigenes obtained annotation with gene descriptions from NR, Swiss-Prot, COG, and KEGG databases. This study demonstrated that the RNA-seq technology could be used as a rapid and efficient method for *de novo *transcriptome analysis of non-model plant organisms that provides a good resource of gene expression data for further analysis. A protein-protein interaction network of expressed genes was constructed in *S. japonica*. The topological analysis revealed that degree correlation of the giant component was disassortative and had small-world properties. This result implied that the protein-protein interaction network in *S. japonica* might have resulted from a long-term evolution to ensure both stability and low energy consumption protein-protein interactions.

## Supplementary Material

Additional file1: Information of unigenes expression in S. japonica.
Additional file2: DNA sequences of all unigenes in S. japonica.
Additional file3: Potential protein coding sequences of all unigenes in S. japonica.
Additional file4: Quality score distribution across all bases.
Additional file5: Quality score distribution over all sequences.
Additional file6: Distribution of unigenes mapped to the speciesshown by pie graph.
Additional file7: Detailed information for GO classification of unigenes in S. japonica.
Additional file8: COG functional classification list of unigenes in S. japonica.
Additional file9: Detailed information for unignenes mapping KEGG pathway in S. japonica.
Additional file10: Illustration of the whole network of unigenes in S. japonica.
Additional file11: Protein-protein interactions of unigenes in S. japonica.

## Figures and Tables

**Table 1:** Summary of sequence assembly by trinity after Illumina sequencing.

	Number	Mean size (bp)	N50 size (bp)	Total nucleotides (bp)
Read	86139654	101	101	8700105054
Unique transcript	143010	1482	1155	211940997
Unigene	57614	901	545	51899592

**Table 2:** Summary of annotation of *S. japonica* unigenes.

Category	Number	Percentage
Nr annotated unigenes	27507	47.74%
Swissprot annotated unigenes	20463	35.52%
GO classified unigenes	15063	26.14%
COG classified unigenes	5863	10.18%
KEGG classified unigenes	2869	4.98%

**Table 3:** The average clustering coefficient (*C*) and shortest path length (*L*) of the giant component of the unigenes of *S. japonica* measured using Erdős-Rényi, Watts-Strogatz, and Barabási-Albert network models.

Item	*C *	*L *
Giant component	4.68*E* − 03	5.01
Erdős-Rényi	1.49*E* − 04	5.84
Watts-Strogatz	2.20*E* − 06	2.00
Barabási-Albert	4.34*E* − 04	3.35

## Abbreviations

UT: Unique transcript
NR: NCBI non-redundant protein
COG: Cluster of Orthologous Groups
GO: Gene ontology
KEGG: Kyoto Encyclopedia of Genes and Genomes database
KAAS: KEGG Automatic Annotation Server
BLAST: Basic Local Alignment Search Tool
PPI: Protein-protein network.
