# Supplementary figures and images for: De Novo Assembly and Characterization of Sophora japonica Transcriptome Using RNA-seq
Source: Biomed Res Int. 2014 Jan 2;2014:750961. doi: 10.1155/2014/750961 (PMC3910276; doi:10.1155/2014/750961)

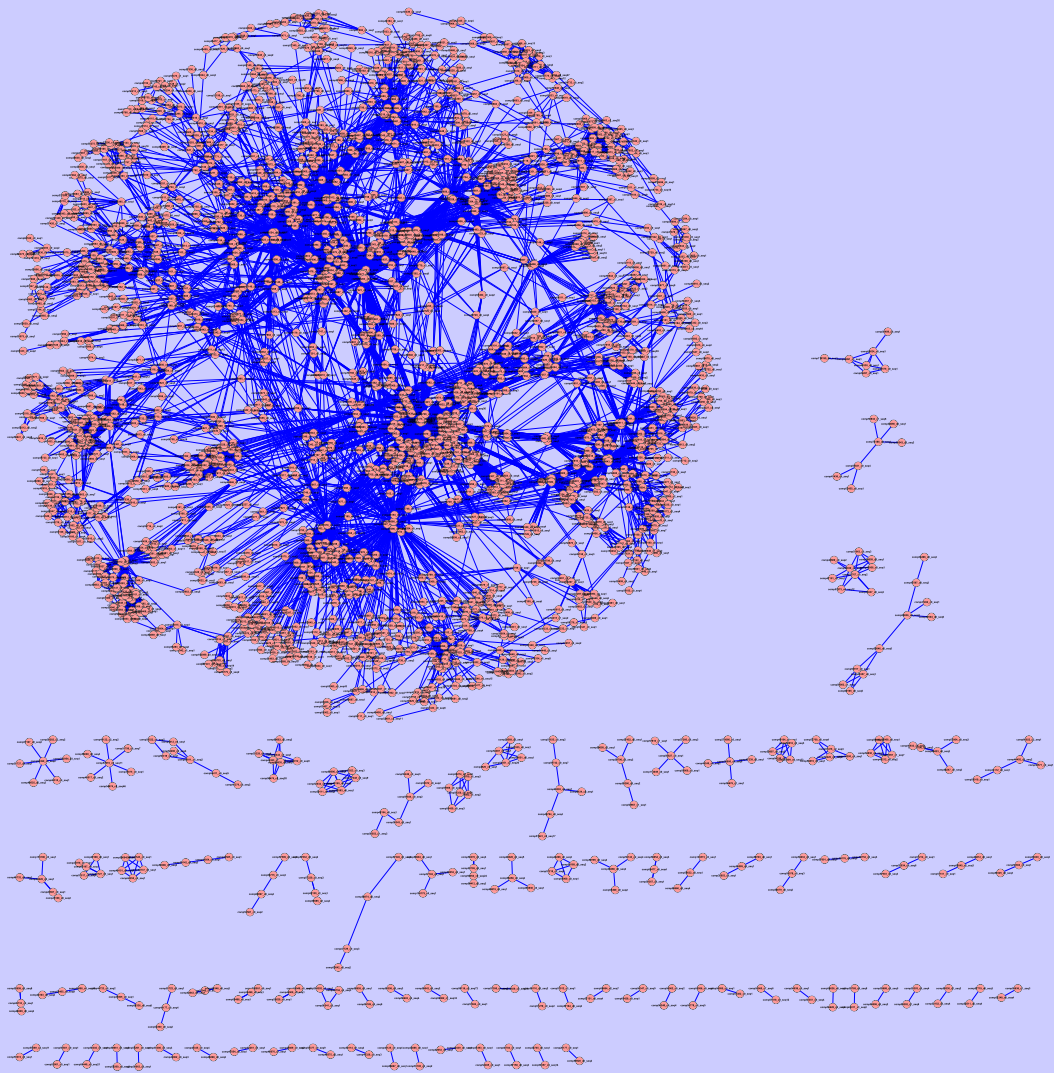

Supplement: Supplementary file 1 — Additional file1: Information of unigenes expression in S. japonica. Additional file2: DNA sequences of all unigenes in S. japonica. Additional file3: Potential protein coding sequences of all unigenes in S. japonica. Additional file4: Quality score distribution across all bases. Additional file5: Quality score distribution over all sequences. Additional file6: Distribution of unigenes mapped to the speciesshown by pie graph. Additional file7: Detailed information for GO classification of unigenes in S. japonica. Additional file8: COG functional classification list of unigenes in S. japonica. Additional file9: Detailed information for unignenes mapping KEGG pathway in S. japonica. Additional file10: Illustration of the whole network of unigenes in S. japonica. Additional file11: Protein-protein interactions of unigenes in S. japonica. [file 750961.f1.zip › 750961.f1/Additional file/Additional file10 visual network.pdf]

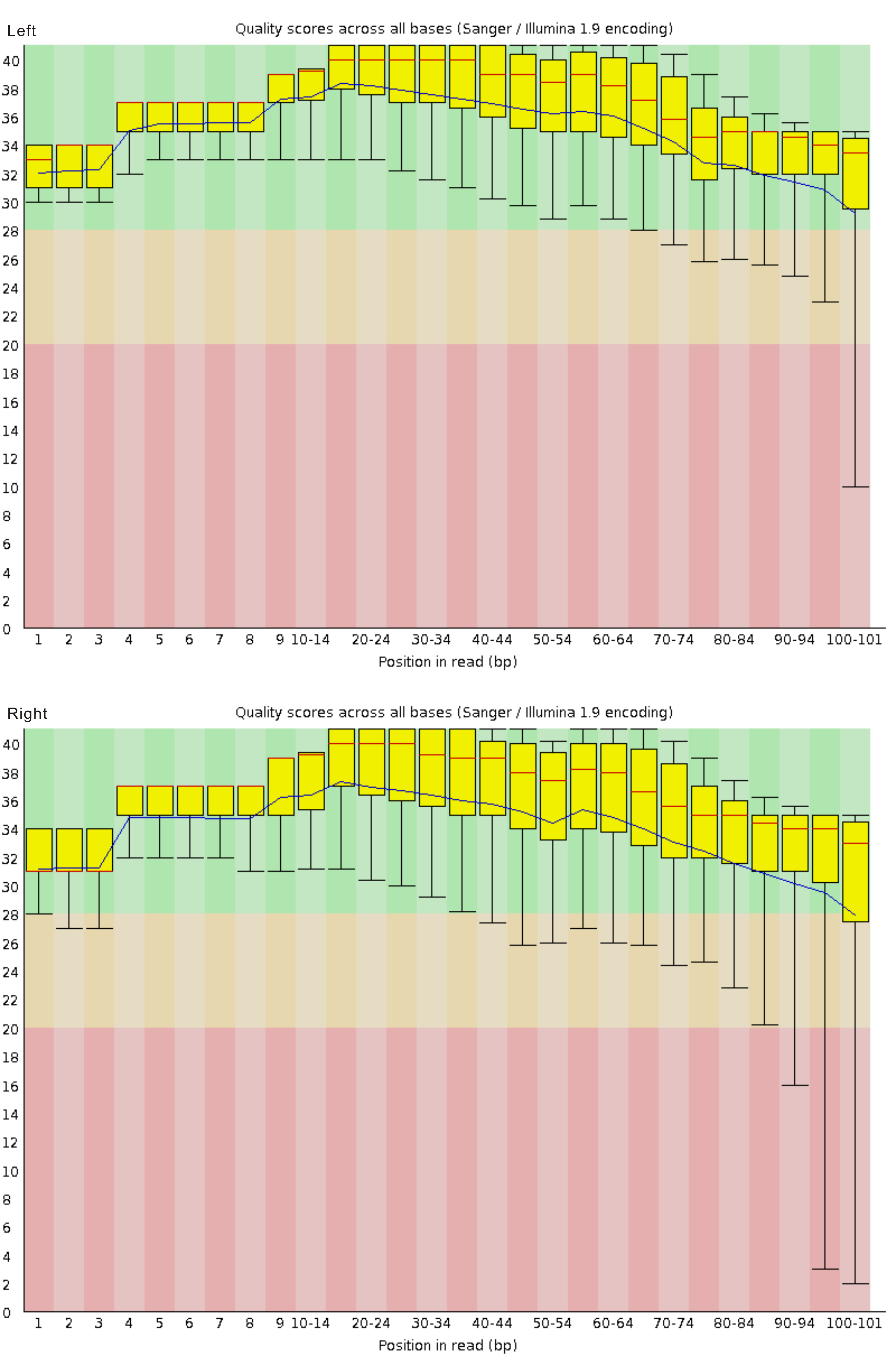

Supplement: Supplementary file 1 — Additional file1: Information of unigenes expression in S. japonica. Additional file2: DNA sequences of all unigenes in S. japonica. Additional file3: Potential protein coding sequences of all unigenes in S. japonica. Additional file4: Quality score distribution across all bases. Additional file5: Quality score distribution over all sequences. Additional file6: Distribution of unigenes mapped to the speciesshown by pie graph. Additional file7: Detailed information for GO classification of unigenes in S. japonica. Additional file8: COG functional classification list of unigenes in S. japonica. Additional file9: Detailed information for unignenes mapping KEGG pathway in S. japonica. Additional file10: Illustration of the whole network of unigenes in S. japonica. Additional file11: Protein-protein interactions of unigenes in S. japonica. [file 750961.f1.zip › 750961.f1/Additional file/Additional file4 quality scores across all bases.png]

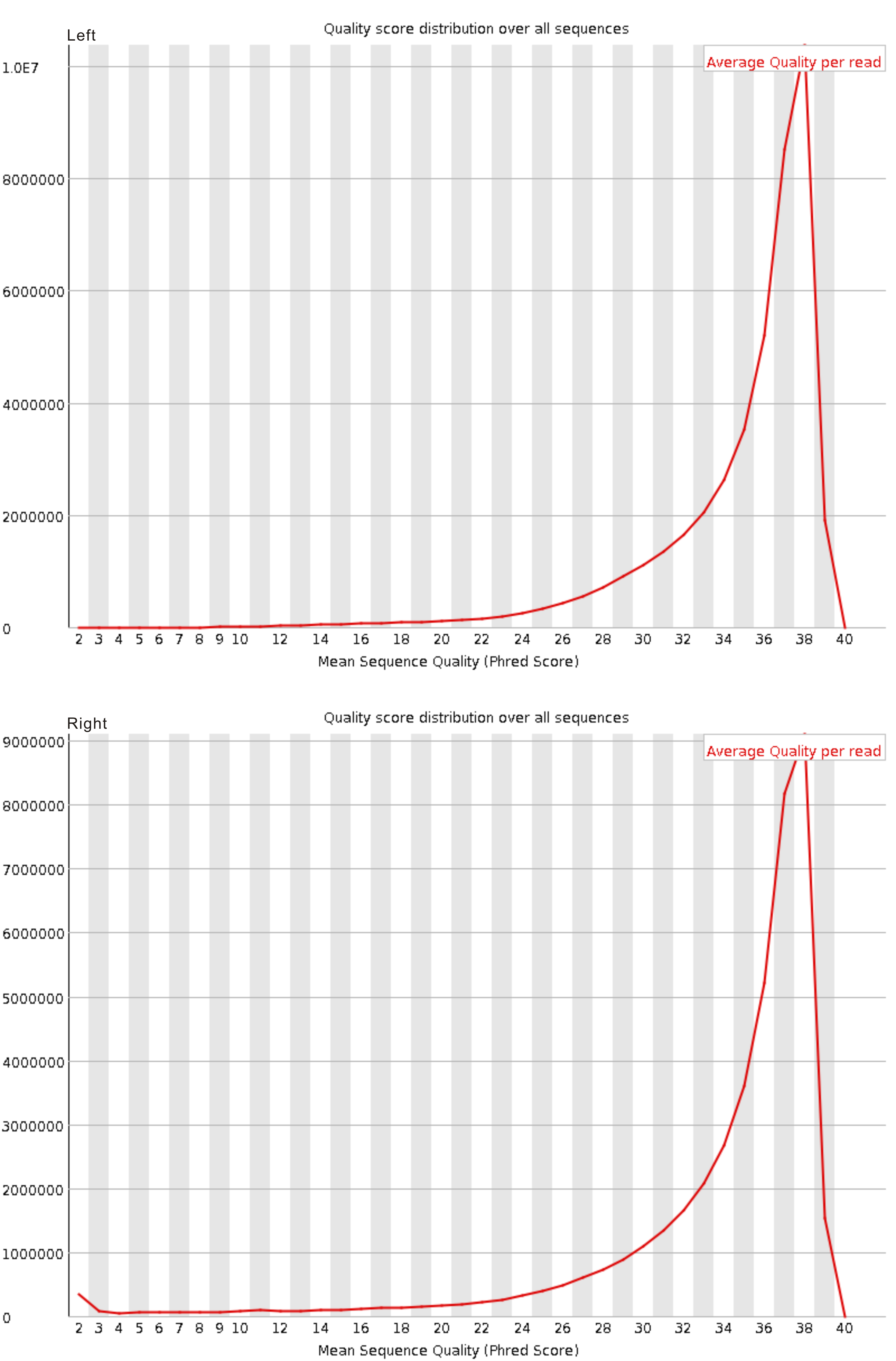

Supplement: Supplementary file 1 — Additional file1: Information of unigenes expression in S. japonica. Additional file2: DNA sequences of all unigenes in S. japonica. Additional file3: Potential protein coding sequences of all unigenes in S. japonica. Additional file4: Quality score distribution across all bases. Additional file5: Quality score distribution over all sequences. Additional file6: Distribution of unigenes mapped to the speciesshown by pie graph. Additional file7: Detailed information for GO classification of unigenes in S. japonica. Additional file8: COG functional classification list of unigenes in S. japonica. Additional file9: Detailed information for unignenes mapping KEGG pathway in S. japonica. Additional file10: Illustration of the whole network of unigenes in S. japonica. Additional file11: Protein-protein interactions of unigenes in S. japonica. [file 750961.f1.zip › 750961.f1/Additional file/Additional file5 per_sequence_quality.png]

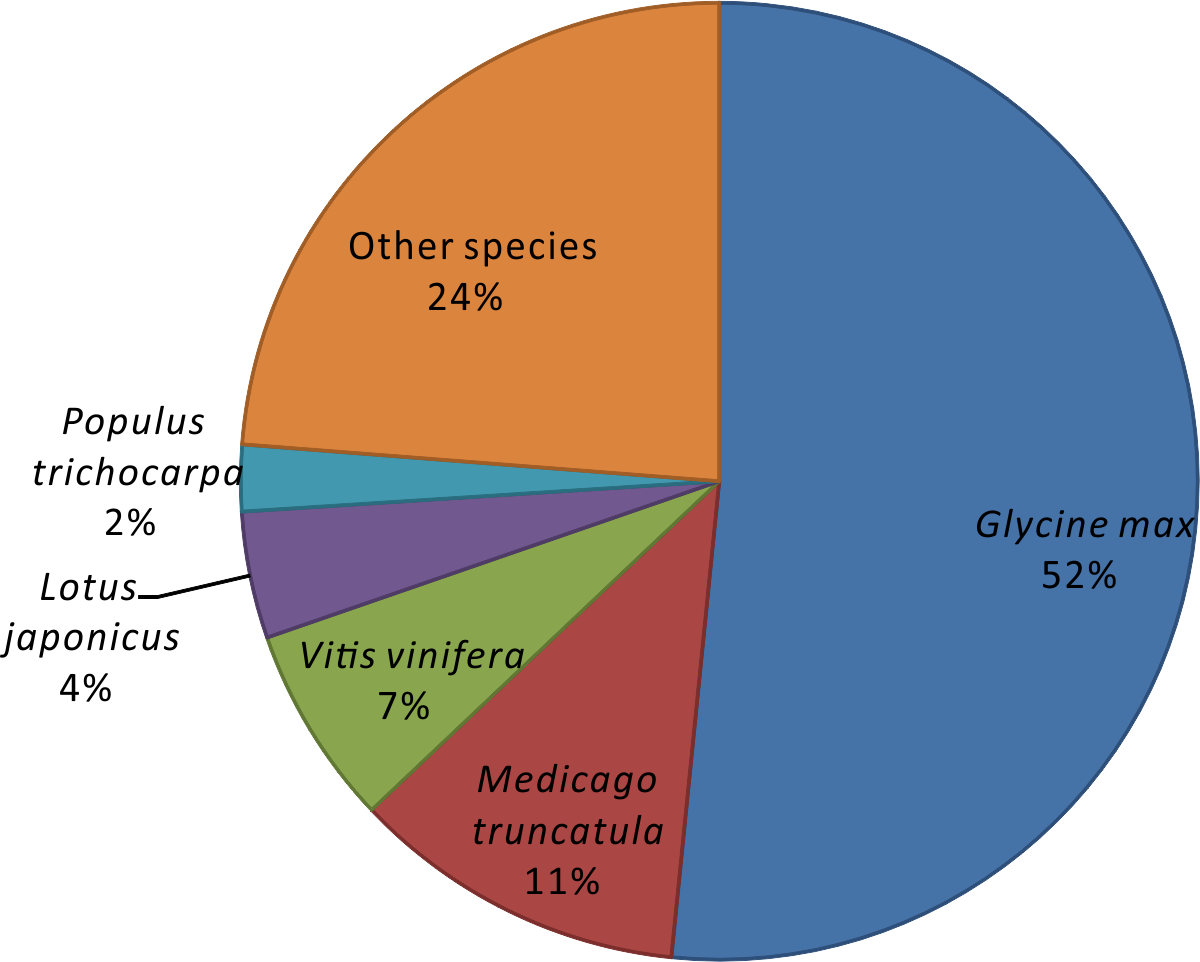

Supplement: Supplementary file 1 — Additional file1: Information of unigenes expression in S. japonica. Additional file2: DNA sequences of all unigenes in S. japonica. Additional file3: Potential protein coding sequences of all unigenes in S. japonica. Additional file4: Quality score distribution across all bases. Additional file5: Quality score distribution over all sequences. Additional file6: Distribution of unigenes mapped to the speciesshown by pie graph. Additional file7: Detailed information for GO classification of unigenes in S. japonica. Additional file8: COG functional classification list of unigenes in S. japonica. Additional file9: Detailed information for unignenes mapping KEGG pathway in S. japonica. Additional file10: Illustration of the whole network of unigenes in S. japonica. Additional file11: Protein-protein interactions of unigenes in S. japonica. [file 750961.f1.zip › 750961.f1/Additional file/Additional file6.png]
